# Advances in artificial intelligence-based microbiome for PMI estimation

**DOI:** 10.3389/fmicb.2022.1034051

**Published:** 2022-10-04

**Authors:** Ziwei Wang, Fuyuan Zhang, Linlin Wang, Huiya Yuan, Dawei Guan, Rui Zhao

**Affiliations:** ^1^Department of Forensic Pathology, China Medical University School of Forensic Medicine, Shenyang, China; ^2^Liaoning Province Key Laboratory of Forensic Bio-evidence Science, Shenyang, China

**Keywords:** postmortem submersion interval, forensic medicine, microbial community, artificial intelligence, microbial succession

## Abstract

Postmortem interval (PMI) estimation has always been a major challenge in forensic science. Conventional methods for predicting PMI are based on postmortem phenomena, metabolite or biochemical changes, and insect succession. Because postmortem microbial succession follows a certain temporal regularity, the microbiome has been shown to be a potentially effective tool for PMI estimation in the last decade. Recently, artificial intelligence (AI) technologies shed new lights on forensic medicine through analyzing big data, establishing prediction models, assisting in decision-making, etc. With the application of next-generation sequencing (NGS) and AI techniques, it is possible for forensic practitioners to improve the dataset of microbial communities and obtain detailed information on the inventory of specific ecosystems, quantifications of community diversity, descriptions of their ecological function, and even their application in legal medicine. This review describes the postmortem succession of the microbiome in cadavers and their surroundings, and summarizes the application, advantages, problems, and future strategies of AI-based microbiome analysis for PMI estimation.

## Introduction

Postmortem interval (PMI) is the time between the discovery and examination of the body and the occurrence of death. Relatively accurate estimation of PMI has always been an important issue in the field of forensic medicine. PMI estimation based on postmortem phenomena is still the common and feasible way in forensic practice. Owing to the inference of PMI being highly susceptible to the individual’s physical condition, cause of death, and environmental conditions, the predicted accuracy of PMI cannot meet the requirements of the actual work. Microbial communities are involved in the decomposition of deceased bodies and present a certain regular succession on the host, making it possible to predict PMI based on the microbial communities (Diez Lopez et al., 2022). In the last decade, postmortem microbiome has been applied to predict PMI, and technologies for microorganisms cover the shortfall of traditional morphological methods. Traditional methods using microbial cultivation of target-specific strains are highly dependent on culture conditions and have limitations for the analysis of the component and function of microbial communities (Cecchini et al., 2012; Zhou and Bian, 2018). Next-generation sequencing (NGS) has brought revolutionary progress to the study of microorganisms in forensic medicine. NGS can quickly and accurately analyze the entire microbial community, including many species that cannot be cultured in the laboratory (Kuiper, 2016). Meanwhile, the use of NGS brings a huge amount of microbial data, which requires an efficient data analysis method to process. Recently, artificial intelligence (AI) technologies shed new lights on forensic medicine through analyzing big data, establishing prediction models, assisting in decision-making, etc. (Geradts, 2018). Importantly, the development of AI techniques has facilitated forensic practitioners to improve understanding of microbial communities through analysis of the postmortem changes of microorganisms in different organs/tissues at various taxonomic levels (Speruda et al., 2021).

This review summarizes the succession patterns of postmortem microbial communities both on cadavers and their surrounding environment, and analyzes the advances of AI techniques on PMI estimation and their potential application on PMI prediction in the future.

## Postmortem microbial succession in cadavers

Microorganisms predominantly colonize five parts of cadavers: the gastrointestinal tract, the oral cavity, skin, the respiratory tract, and the genitourinary tract. Due to the convenience of sampling from living individuals, most studies have focused on the gastrointestinal tract, the oral cavity, and skin (Dash and Das, 2022). In recent years, numerous studies have been conducted on the succession pattern of microbial communities and PMI prediction based on different organs in both human remains and animal models. There are dramatic postmortem changes of microbial community succession in different organs (Pechal et al., 2014; DeBruyn and Hauther, 2017; Dash and Das, 2022) The diversity of most microorganisms shows similar decreasing trends with PMI, presenting a significant negative linear correlation (Pechal et al., 2014; DeBruyn and Hauther, 2017; Li et al., 2020). At the phylum level, *Proteobacteria* and *Firmicutes* dominate the microbial communities in different postmortem organs in both terrestrial and water environments, making them potential markers for PMI or postmortem submersion interval (PMSI) prediction (Benbow et al., 2015; He et al., 2019; Javan et al., 2019; Yuan et al., 2020; Dash and Das, 2022). The detailed taxonomy on families or genus levels of *Proteobacteria* and *Firmicutes* would undoubtedly enhance understanding of postmortem microbial community succession in different samples. For instance, in terrestrial conditions, Tuomisto et al. (2013) found that the pericardial fluid and liver remain sterile within 5 days postmortem, while the highest abundances of *Bifidobacteria*, *Bacteroides*, *Enterobacter*, and *Clostridia* are detected in the liver, mesenteric lymph node, pericardial fluid of cadavers within 7 days, providing a short-term PMI fetch reference. Some studies revealed changes of dominant microorganisms in different human organs and blood specimens after death. *Bacillus* and *Lactobacillus* predominated in the short-term after death followed by an exponential decrease with the extension of PMI, while parthenogenic anaerobic bacteria, such as *Clostridium*, were predominant in the late phase of PMI (Can et al., 2014; Hauther et al., 2015; Javan et al., 2016; DeBruyn and Hauther, 2017). This accounted for the phenomenon of Postmortem Clostridium Effect (PCE) at decomposition stage (Javan et al., 2017). In addition, the alterations in several species of *Clostridium* may provide more information on different stages of PMI, for example, *C. novyi* was relatively more abundant in late PMI; however, an unknown member of the genus *Clostridium* was found to be more abundant in early PMI (Javan et al., 2016). In consistent with the data in terrestrial conditions, some studies demonstrated that *Enterococcus* and *Clostridium* were predominated on the skin and bones of water-dead pigs in the late stage of PMI (Benbow et al., 2015; Cartozzo et al., 2021). Our recent studies also showed that *Clostridium* in the lung and cecum were associated with PMSI in the fresh water environment (Wang et al., 2020; Zhang et al., 2022).

Numerous studies revealed the influence of different factors on PMI estimation, such as sample type (Javan et al., 2019; Lutz et al., 2020) and environmental factors (Iancu et al., 2018; Diez Lopez et al., 2022). Furthermore, the effects of gender on the analysis of microbial communities cannot be ignored, with evidence that the genera *Rothia* and *Streptococcus* were only present in the visceral organs of men, while an abundance of the genera *Clostridium* and *Pseudomonas* were found in a higher proportion of heart tissues from women compared with those from men (Javan et al., 2016; Bell et al., 2018). The study of sex-specific microbial communities could help to improve the precise of PMI estimation. Considering so many factors that affect postmortem microbial community succession and PMI estimation, exploration of an effective detection method and sufficient microbial datasets should be undertaken in future work.

## Postmortem microorganisms changes in the surrounding environment

Microbial communities of cadavers interact with the surrounding environment. Although microbial community succession in carcasses placed on different soil types tends to be consistent postmortem (Metcalf et al., 2016), the microbial community in the environment does affect the process of decomposition. For example, mice that interact with normal soil decompose faster than the cadavers placed on sterile soil (Lauber et al., 2014). In addition, decomposed cadavers release various adipose tissues, volatile fatty acids, organic acids, organic nitrogen, and bacterial flora—such as obligately anaerobic *Bacteroides*—into the soil (Vass et al., 1992). This is followed by changes in the microbial community in the soil after death, which make it possible to predict PMI based on soil microbes (Cobaugh et al., 2015).

Terrestrial soil microbes related to forensic research can be broadly divided into surface soil and buried soil (Carter et al., 2007). Surface soil microbial communities exhibit decreased trends in abundance, diversity, and evenness during decomposition, with a sharp increase in the abundance of *Firmicutes* and *Proteobacteria* and a decrease in the abundance of *Acidobacteria* in soils around cadavers (Cobaugh et al., 2015; Adserias-Garriga et al., 2017a,b; Procopio et al., 2019). In contrast, a study found that buried soil microbial communities showed the trends of increasing abundance, decreasing evenness, and consistent diversity, and the microbial composition remained unchanged throughout the decomposition process, with *Proteobacteria* being the most abundant phylum (Finley et al., 2016). According to the microbial community succession of soils surrounding cadavers, Procopio et al. (2019) revealed that *Bacteroides* spp., specific mammal-derived taxa, could be detected in the buried soil 6 months after PMI. However, soil microbial communities are easily influenced by environmental factors (Chernov and Zhelezova, 2020), such as temperature, moisture, vegetation cover, and insect activity. Habtom et al. (2019) analyzed different soils in five different rainfall zones and found significant differences in bacterial population structure among soil types in the same geographic location. Yang et al. (2021) studied the variation of microbial community composition in 529 soil samples from 61 urban districts of 10 cities in China at a large spatial scale and showed that the similarity of urban soil bacterial communities decreased significantly with increasing geographical distance. Although the population structure of soil bacteria within the same city was relatively similar, the identification accuracy of random soil samples was 90.0% at the city level and 66.7% at the district level within the city. However, the use of distinguished soil microorganisms in forensic science needs to be confirmed in further studies. Owing to the inherent microbial communities in different soils, it is difficult to compare the microbial databases from numerous studies using different soils for PMI prediction. Hence, predicting PMI according to the soil microbial community succession alone is inadequate; a better option would be to combine the soil microbial community with that in cadavers and consider the influence of entomology and ecology.

## Application of artificial intelligence for PMI prediction based on microbial data

Improvements in sequencing technology, especially NGS technology, provide sufficient genomic information for analyzing entire microbial communities (Kuiper, 2016). However, owing to the massive amount of data generated and statistical validity, an effective analysis method aligned to digging deeper is needed. AI has the advantages of effective assessment models by comprehensively examining and mining multidimensional big data, evaluating weights, and identifying patterns of data changes to establish an effective “time fingerprint” mathematical model (Zou et al., 2020). Hereafter, the presented studies on PMI prediction using NGS technology are predominantly based on AI.

Postmortem microbiome analysis for PMI estimation has been improved to a relatively accurate stage using AI. At present, Machine Learning (ML) is the main AI technology used in forensic studies, ML is one type of artificial intelligence that develops algorithms to enable computers to learn from existing data without explicit programming (Zaharchuk et al., 2018). Such ML methods include k-nearest neighbor (KNN), Partial Least Squares (PLS), random forest (RF), support vector machine (SVM), and artificial neural network (ANN; Table 1). For instance, Johnson et al. (2016) and colleagues constructed a KNN model (*k* = 4) for PMI estimation using microbial communities from skin in the nasal cavity and ear canal, which developed an error of only 55 accumulated degree hours (ADD) over a time period of 800 ADD. Cao et al. (2021) used segmented cecum microbial community data from rats to construct PLS models and found that the PLS model was effective in the first 9 days after death. RF is the most common ML algorithm in microbial community studies for PMI prediction and has the advantages of strong learning ability, robustness, and feasibility of the hypothesis space (Ao et al., 2019). In the terrestrial environment, Metcalf et al. (2013) established a RF regression model for the first time based on the microbial community in mouse cadaver skin and abdominal cavity samples, and this model predicted PMI with a mean absolute error (MAE) of 3.30 ± 2.52 days within the first 34 days and further provided the concept of “microbial clock.” Subsequently, RF regression models were constructed using microbial communities from dead pig skin and oral swabs for PMI predictions, and the accuracy was up to 94.4% within 5 days postmortem (Pechal et al., 2014). Zhang et al. (2021) compared the separate RF regression models using microbial communities from different organs and buried soils and found that the lowest MAE value was for buried soils within 60 days after death. Zhao et al. (2022) and colleagues used rat oral microorganisms to construct a RF model, and the R^2^ of the model within 59 days was 93.94%. In the aquatic environment, our recent studies provide evidence that RF regression models were effective for predicting PMSI based on the microbiota succession of the mouse cecum, with a MAE of 0.818 days within the 14 days postmortem (Zhang et al., 2022). For long-term aquatic environmental decomposition (>1 year), different researchers constructed RF regression models to predict PMSI using microbial communities of porcine ribs and scapula. The model using rib microbiota performed best within 353 days, with a root mean square error (RMSE) of ±27 days, while the model using scapula microbiota performed best within 579 days with a RMSE of ±63 days (Cartozzo et al., 2021; Randall et al., 2021). Kaszubinski et al. (2022) constructed a RF regression model using microbial communities from pig bone within 547 days, and the model exhibited high accuracy, explaining more than 80% of the variation in PMSI. Recently, Liu et al. (2020, 2021) compared the performance of RF, SVM, and ANN models using microbial communities in cecum and concluded that the ANN model performed best, with a MAE of 1.5 ± 0.8 h within 24 h and 14.5 ± 4.4 h within 15 days after death for PMI prediction. These findings suggested the combination of multiple AI methods might improve the accuracy of PMI estimation.

**Table 1:** Application of AI on microbiome for predicting PMI.

Animal model	Experimental environment	PMI/PMSI	AI model	Model performance	Sampling location	References
Human	Temperate forest	800ADD	KNN	MAE ±55ADD	Nasal cavity, Ear canal	Johnson et al. (2016)
Rat	Artificial climate chamber	30d	PLS	RMSE within 9d: 1.96d	Cecum	Cao et al. (2021)
				RMSE 12d later: 5.37d		
				RMSE within 30d: 6.57d		
Mice	Laboratory	48d	RF	MAE 3.30+/−2.52d	Skin	Metcalf et al. (2013)
Pig	Temperate forest	5d	RF	94.4% accuracy rate	Skin, Oral cavity	Pechal et al. (2014)
Rat	Gravesoil	60d	RF	MAE 1.82d	Gravesoil	Zhang et al. (2021)
				MAE 2.06d	Rectum	
				MAE 2.13d	Skin	
Rat	Sterile anti-scavenging cages	59d	RF	R2 93.94%	Oral cavity	Zhao et al. (2022)
Porcine bones	Natural fresh river	353d	RF	RMSE±27d	Rib	Cartozzo et al. (2021)
				RMSE±29d	Scapulae	
Porcine bones	Freshwater lake	579d	RF	RMSE±104d	Rib	Randall et al. (2021)
				RMSE±63d	Scapulae	
Sus scrofa	Freshwater pond	547d	RF	>80% variation explained	Bone	Kaszubinski et al. (2022)
Mice	Artificial climate chamber	15d	RF	MAE 20.01 h	Cecum	Liu et al. (2021)
			ANN	MAE Within 24 h: 1.5 ± 0.8 h, Within 15d: 14.5 ± 4.4 h		Liu et al. (2020)

**Figure 1:**
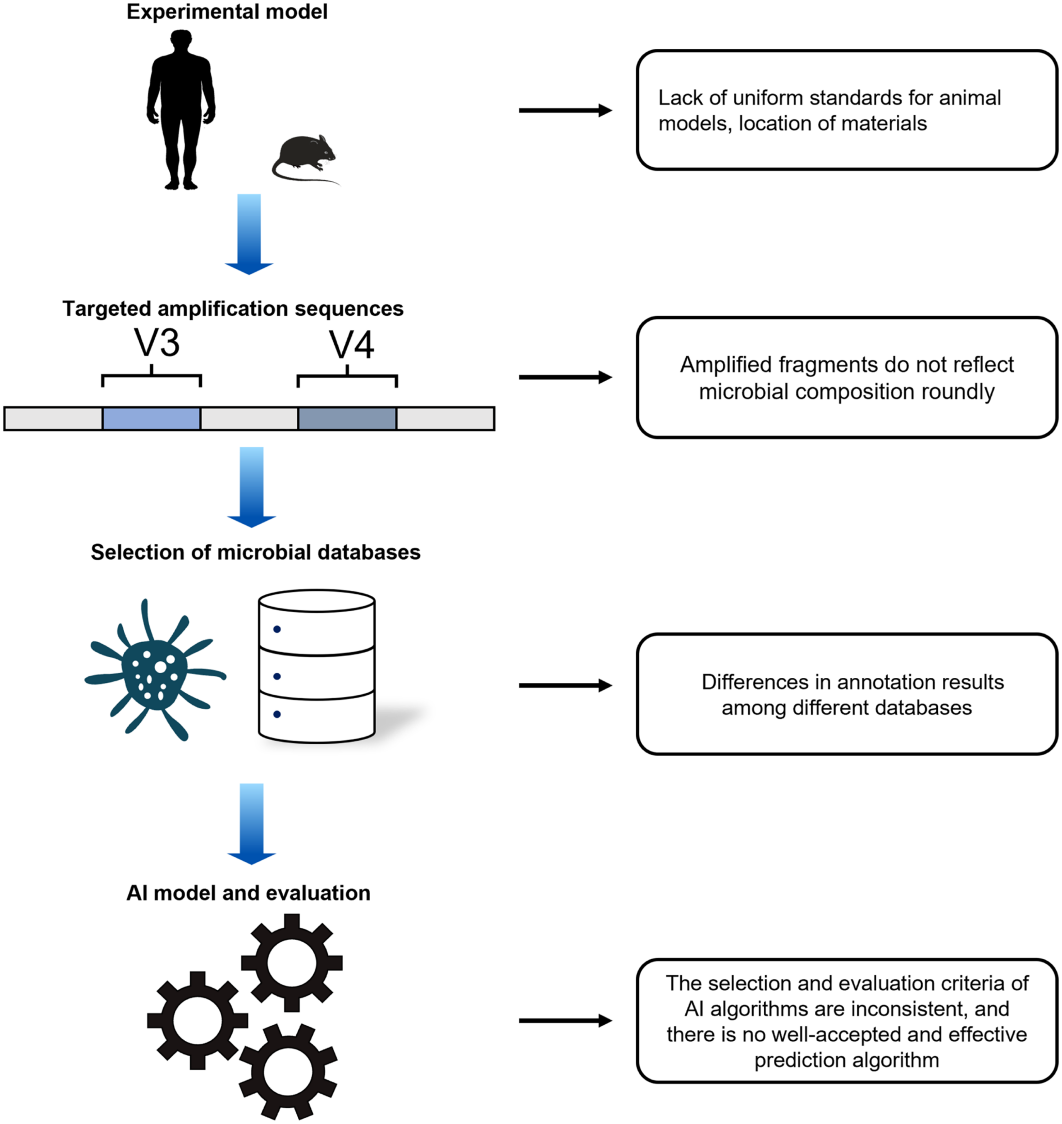
Problems for AI prediction of PMI.

Although many exciting results have been achieved to date to prove that microbial communities combined with AI are potentially effective tools for predicting PMI, there are still many problems with using AI analysis of microbiological data to study PMI (Figure 1). First, there is lack of unified standardization in experimental models, collected samples, and data analysis, which means the predicted results of PMI are not credible for the courtroom (Diez Lopez et al., 2022). Many complex environmental and artificial factors can potentially affect the succession of microorganisms. Second, NGS has the limitation of short reads and low accuracy of species identification (Yakun et al., 2019); consequently, most studies have predominantly targeted amplification of the V3 and V4 regions of the 16S rRNA gene, and these fragments only provide an approximate picture of the bacterial phyla (Verma et al., 2018). Accurate detailed taxonomy annotation of microorganisms requires full-length amplification of DNA. In addition, more advanced methods to disclose all microbial community species are needed. A recent study started to use third-generation sequencing technology for microbial research (Wang et al., 2021). Third, the main microbial datasets (Silva, Greengenes) for forensic PMI studies were mainly established based on clinical or environmental studies (Quast et al., 2013; Balvočiūtė and Huson, 2017). These datasets contain different numbers and types of microbial species, which could result in differences in annotation even when using the same sequencing data. Finally, the black box and uncertainty are central challenges in designing AI tools (Saffiotti, 1987). Although AI techniques are widely used for PMI estimation, the different predicted models for PMI present difference in estimated effectiveness, especially using detailed taxonomic levels, such as species and genera. Consequently, it is necessary to explore a well-recognized AI method for its application in forensic medicine (Metcalf, 2019).

## Future outlook

The widespread use of AI provides new insights into forensic PMI estimation. However, current advances in AI techniques using the microbiome for PMI prediction highlight three key points to improve the accuracy of PMI studies in the future.

1. The establishment and development of microbiome biobanks for forensic purposes are necessary. Considering the complex influences of models, samples, locations, environmental factors, and postmortem intervals, forensic researchers should collaborate to pool raw data and construct a microbiome biobank for forensic purposes.
2. Deep learning (DL) may shed new light on accurate predicted models for PMI. DL are considered one of the cutting-edge areas of development and study in almost all scientific and technological fields and has allowed for resolving many challenges faced by standard ML algorithms. The basis of DL is often implicated in neural network systems, where they are used to create systems that have the capability to complete complex data recognition, interpretation, and generation (Rahaman et al., 2020). AI techniques for analyzing microbiota data are still in their infancy because the amount of data used in most studies is still too low to meet the demands of DL. Deep learning—which allows neural networks to learn how to capture features by themselves (Cheng et al., 2018)—will enhance the accuracy of AI models for PMI prediction.
3. AI technologies for multi-omics provide a future direction for PMI estimation. Although microbiome analysis with AI has been shown to be effective for predicting PMI, integrated omics—including microbiomes, metabonomics, transcriptomics, and proteomics—will further improve the accuracy of PMI inference with the development of AI techniques.

## Author contributions

RZ and DG designed the manuscript and edited the manuscript. RZ and ZW wrote the manuscript. FZ, LW, and HY searched, edited, and reviewed the literature. All authors have read and commented on the manuscript.

## Funding

This research was supported by the National Natural Science Foundation of China (grant numbers: 81971793 and 81772023), Shenyang Science and Technology Innovation Support Plan for Young and Middle-aged Talent (grant number: RC200412), the Natural Science Foundation of Liaoning Province (2022-YGJC-74), and National Key Research and Development Program of China (grant number: 2022YFC3302002).

## Conflict of interest

The authors declare that the research was conducted in the absence of any commercial or financial relationships that could be construed as a potential conflict of interest.

## Publisher’s note

All claims expressed in this article are solely those of the authors and do not necessarily represent those of their affiliated organizations, or those of the publisher, the editors and the reviewers. Any product that may be evaluated in this article, or claim that may be made by its manufacturer, is not guaranteed or endorsed by the publisher.
